# Effects of grapefruit, grapefruit juice and water preloads on energy balance, weight loss, body composition, and cardiometabolic risk in free-living obese adults

**DOI:** 10.1186/1743-7075-8-8

**Published:** 2011-02-02

**Authors:** Heidi J Silver, Mary S Dietrich, Kevin D Niswender

**Affiliations:** 1 Department of Medicine, Division of Gastroenterology, Hepatology and Nutrition, Vanderbilt University School of Medicine, Vanderbilt University, Nashville, TN 37232, USA; 2 Department of Biostatistics, Vanderbilt University Schools of Nursing and Medicine, Vanderbilt University, Nashville, TN 37232, USA; 3 Department of Medicine, Division of Diabetes, Endocrinology and Metabolism, Vanderbilt University School of Medicine, Vanderbilt University, Nashville, TN 37232, USA; 4 Department of Veterans Affairs, Tennessee Valley Healthcare System, TN, USA

## Abstract

**Background:**

Reducing dietary energy density has proven to be an effective strategy to reduce energy intakes and promote weight control. This effect appears most robust when a low energy dense preload is consumed before meals. Yet, much discussion continues regarding the optimal form of a preload. The purpose of the present study was to compare effects of a solid (grapefruit), liquid (grapefruit juice) and water preload consumed prior to breakfast, lunch and dinner in the context of caloric restriction.

**Methods:**

Eighty-five obese adults (BMI 30-39.9) were randomly assigned to (127 g) grapefruit (GF), grapefruit juice (GFJ) or water preload for 12 weeks after completing a 2-week caloric restriction phase. Preloads were matched for weight, calories, water content, and energy density. Weekly measures included blood pressure, weight, anthropometry and 24-hour dietary intakes. Resting energy expenditure, body composition, physical performance and cardiometabolic risk biomarkers were assessed.

**Results:**

The total amount (grams) of food consumed did not change over time. Yet, after preloads were combined with caloric restriction, average dietary energy density and total energy intakes decreased by 20-29% from baseline values. Subjects experienced 7.1% weight loss overall, with significant decreases in percentage body, trunk, android and gynoid fat, as well as waist circumferences (-4.5 cm). However, differences were not statistically significant among groups. Nevertheless, the amount and direction of change in serum HDL-cholesterol levels in GF (+6.2%) and GFJ (+8.2%) preload groups was significantly greater than water preload group (-3.7%).

**Conclusions:**

These data indicate that incorporating consumption of a low energy dense dietary preload in a caloric restricted diet is a highly effective weight loss strategy. But, the form of the preload did not have differential effects on energy balance, weight loss or body composition. It is notable that subjects in GF and GFJ preload groups experienced significantly greater benefits in lipid profiles.

**Trial registration:**

ClinicalTrials.gov NCT00581074

## Background

As the clinical and economic burden of obesity grows [1], practical interventions for weight management offer considerable therapeutic and cost containment advantages. Dietary strategies range from restricting calories, manipulating macronutrient composition or enhancing single nutrients, to altering energy density. Accumulating evidence indicate that reducing dietary energy density (kilocalories per gram of food) increases satiety and decreases energy intake [2-4]. This effect appears most robust when a low energy dense preload is consumed before meals. For example, women who consumed a low energy dense soup preload rated their hunger and prospective food consumption significantly lower and consumed 26% fewer calories in subsequent meals [5]. In another experiment, women reported feeling more full and consumed 7-12% less calories from lunch after a low energy dense salad [6]. While some studies indicate that solids have greater effects on reducing food and energy intake [7,8], others demonstrate that liquids can be as effective [5]. Thus, the evidence on the optimal form of a preload, i.e. solid, semi-solid or liquid, remains inconclusive [9,10].

While it appears that the water content of the item predominately determines its energy density and effects on intakes [11], few studies have been conducted with foods that have naturally high water contents - like fruit. Fruit is also informative because it is readily available in solid, semi-solid and liquid forms. A series of experiments demonstrated significantly less hunger and greater satiety after consuming whole apple, orange and grape compared to apple, orange and grape juice [12]. Further, when matched by energy density, whole apple reduced lunch meal energy intakes more than apple sauce and juice [13].Yet, all three forms reduced lunch meal energy intakes compared to no preload. Notably, the above studies were conducted with healthy normal-weight adults. When lean and obese adults were included, the three forms of apple elicited different appetite ratings, but energy intakes did not differ [14].

The present study was designed to compare the effects of consuming solid and liquid forms of a fruit preload on energy balance, body weight and composition, and cardiometabolic risk factors in free-living obese adults who were prescribed caloric restriction. We chose grapefruit as the preload because grapefruit (GF) and grapefruit juice (GFJ) have high (~91%) water contents. Moreover, consumption of GF and GFJ has been widely publicized in the lay media as an effective strategy for achieving weight loss for over four decades [15]. To rigorously compare preload forms, we matched GF and GFJ preloads by weight, calories, water content, and energy density. In addition, GF and GFJ preloads were compared to a water preload matched by weight (as water has no calories or energy density).

Since dietary fiber content should reduce energy intake by slowing gastric emptying and inducing early satiety [16], we hypothesized that subjects consuming GF preloads would experience greater weight loss due to the potential combined effects of low energy density and higher fiber content. We further hypothesized that subjects consuming the GFJ preload would experience greater reductions in cardiometabolic risk due to the potential combined effects of low energy density with higher bioflavonoid content, which is associated with influencing lipoprotein dynamics [17].

## Methods

### Subject recruitment and enrollment

**Figure 1:**
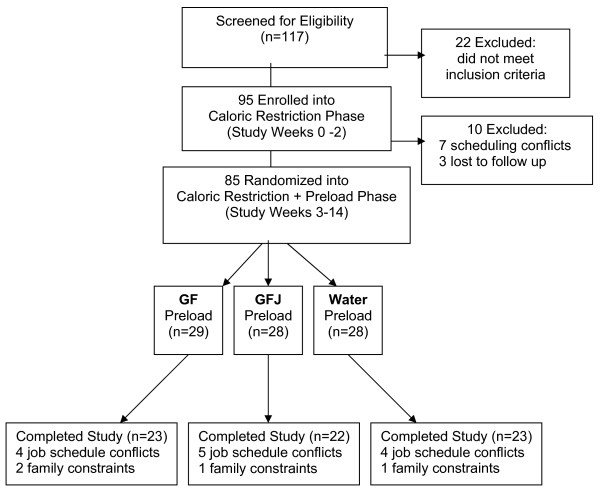
**Flow diagram of study subjects from eligibility criteria screening to study completion**. GF = grapefruit; GFJ = grapefruit juice.

Adults aged 21 to 50 years who responded to print and electronic advertisements were screened by telephone to exclude diabetes, cardiovascular, liver or kidney disease; medications for estrogen replacement, thyroid disease, depression, gastrointestinal disorders; medications metabolized by the cytochrome P450 (CYP) 3A4 enzyme [18]; orexigenic agents; and food allergies or medically restricted diets. The Vanderbilt University Institutional Review Board approved the study protocol which was registered in the U.S. National Institutes of Health ClinicalTrials.gov registry (NCT00581074). The study opened for accrual in March 2006 and enrollment closed in January 2007. One hundred seventeen individuals were scheduled for further eligibility assessment by Registered Dietitians (RD) trained in anthropometry [19] and the U.S. Department of Agriculture multi-pass 24-hour diet recall methodology [20]. Written informed consent was obtained at the enrollment visit (Figure 1).

At the enrollment visit, RDs obtained demographic information and diet, weight and gastrointestinal health history. They administered the Eating Attitudes Test (EAT-26) [21], the Three Factor Eating Questionnaire (TFEQ) [22], the Physical Activity Readiness Questionnaire (PAR-Q) [23], and the Modified Baecke Physical Activity Questionnaire [24]. BMI was assessed by measuring subjects' height (± 0.1 cm) using a wall-mounted stadiometer (SECA 216, Medical Express, Beaverton, OR) and weight (± 0.1 kg) on a digital platform scale (Detecto 8437, Webb City, MO) after subjects removed over-garments, shoes and emptied pockets. Waist and hip circumference (± 0.1 cm) were measured by positioning a flexible measuring tape above the right iliac crest and at the full extension of the buttocks, respectively.

Subjects were included if BMI was 30-39.9 kg/m^2 ^and body weight was under 300 pounds (DEXA table weight limit). Additional exclusions were: weight change of >5 pounds within 3 months, bariatric surgery, disordered eating (EAT-26 score ≥20), non-restrained eating (TFEQ score <14), "yes" to PAR-Q questions, serum triglyceride or LDL-cholesterol level >200 mg/dL, abnormal liver enzyme level, tobacco use, illicit drug use, alcohol intake >1 drink per day, pregnancy (by serum beta-HCG level) or lactation.

Ninety-five subjects who met eligibility were enrolled and instructed to maintain stable body weight by consuming their habitual diet until the first clinic visit. They were trained to use two-dimensional food portion estimation posters (2D Food Portion Visual, Nutrition Consulting Enterprises, Framingham, MA) and measuring utensils to quantify dietary intakes. Between enrollment and the first clinic visit, RDs conducted unannounced telephone-administered diet recalls to capture 24-hour intakes on two nonconsecutive weekdays and one weekend day determined by a computer-generated randomization scheme.

### Caloric restriction phase

At the first clinic visit, subjects were prescribed a diet plan providing a 12.5% calorie restriction compared to individual average baseline energy intakes. The macronutrient composition complied with the Acceptable Macronutrient Distribution Ranges of 30% fat, 50% carbohydrate and 20% protein [25]. Meal plans and sample menus were designed by distributing calorie and macronutrient prescription into 3 meals and 3 snacks daily using Exchange Lists [26]. The number of servings for each food group complied with the *Dietary Guidelines for Americans*, 2005. RDs demonstrated meal plan portions using Life/form^® ^food models (NASCO, Fort Atkinson, WI). Since meal plans included 3 fruit servings daily, subjects agreed to avoid consuming GF or GFJ during the next two weeks. Subjects also avoided taking dietary supplements throughout the study. As responses to the Baecke questionnaire indicated subjects were sedentary, they were also instructed to maintain usual activities and wear pedometers (Accusplit Eagle 120XL, HRM USA, Levittown, PA) to count steps walked daily. Before scheduling individual counseling sessions for the end of study weeks 1 and 2, RDs demonstrated how to complete daily diet, exchange list and pedometer logs.

### Caloric restriction + preload phase

Of the 95 enrolled subjects, 85 submitted logs indicating meal plan compliance during the 2-week caloric restriction phase. These 85 were randomized in an open-label, parallel-arm design to one of three preload conditions for the next 12 weeks. The GF group was instructed to consume 1/2 grapefruit (Florida lot 4281, size 36, 256 g unit weight) before breakfast, lunch and dinner. They were trained to cut, peel and portion GF to exclude only the rind. The GFJ group was trained to portion 127 g GFJ (Ocean Spray unsweetened 100% white GFJ) in pre-measured plastic drinking cups. The GF and GFJ preloads were matched for energy density by weight, kilocalories, water and vitamin C contents, but GF provided more fiber and GFJ more bioflavonoid (Table 1). The water group was trained to portion 127 g of bottled water (Nestlé Pure Life, Greenwich, CT) in pre-measured cups. Subjects were instructed to consume preloads entirely starting 20 minutes before meals [27]. During the caloric restriction + preload phase, meal plans for GF and GFJ groups were adjusted by substituting the GF or GFJ preloads for the three daily fruit servings. At weekly clinic visits, RDs collected empty GF, GFJ and water containers, obtained weight and blood pressure, reviewed logs and counseled subjects to facilitate diet adherence, and distributed preload supplies.

**Table 1:** Preload Properties *

	Weight	Energy	Energy Density	Water	Vitamin C	Fiber	Naringin**
	(g)	(kcal)	(kcal/g)	(g)	(mg)	(g)	(mg)
Grapefruit	128	42	0.331	115.8	42.3	1.13	27.1
Grapefruit Juice	127	46	0.370	115.0	48.3	0.13	39.6
Water	127	0	0	127.0	0	0	0

*The amounts of each variable are based on averaging several pieces of fruit and juice using values obtained from NDS-R, Bowes & Church's [34], and data provided by the State of Florida Department of Citrus.

**Naringin content was chosen to represent bioflavonoid content as it comprises the majority of total flavanones in GF and GFJ, and has been associated with changes in cardiometabolic risk factors. The Davis spectrophotometric method [66] was used to determine flavanones contents.

### Clinical testing

Subjects were scheduled for testing at the Vanderbilt Clinical Research Center (CRC) at baseline (study week 0) and on the days immediately following completion of the caloric restriction phase (study week 2) and caloric restriction + preload phase (study week 14). They were instructed to avoid alcohol and excessive caffeine intake the day before the CRC, and fast from 9:00 pm until arrival at 7:00 am. After weight and vital signs were obtained, visual analog scales (VAS) were administered for subjects to rate hunger, thirst, satiety (amount that could be consumed), appetite (desire for food) and fullness by marking "x" on a 100-mm line anchored with extremes such as "nothing at all" and "an extremely large amount" [28]. For measurement of resting energy expenditure (REE), subjects laid supine, room lights were dimmed, and subjects were habituated to breathing under the canopy in thermoneutral conditions. REE was assessed using a portable metabolic cart system (Medical Graphics CPX Ultima, St. Paul, MN) when oxygen consumption (VO_2_) and carbon dioxide production reached a 30-minute steady state where average change in minute VO_2 _was ≤10% and respiratory quotient ≤5%. Average REE was calculated via the Weir equation [29] with BreezeSuite software (version 6.1B). Dual energy x-ray absorptiometry (DEXA) was performed by a certified densitometrist using a Prodigy whole body scanner (software version 4.3e, Lunar Corp., Madison, WI) to obtain total and regional fat mass, lean mass and bone mineral content with CVs <2.0%. Lastly, subjects performed a timed 400 meter walk to assess exercise capacity [30].

### Biochemical analysis

Standard assays at the Vanderbilt Department of Pathology Clinical Laboratory were performed for lipid profile (triglyceride and total, LDL and HDL-cholesterol) by selective enzymatic hydrolysis, liver function tests (ALT, AST and alkaline phosphatase) by colormetric rate determination, serum glucose by colorimetric timed endpoint method, and insulin by chemiluminescent immunoassay. Presence of metabolic syndrome was defined as ≥ 3 of 5 National Cholesterol Education Program Adult Treatment Panel III criteria [31]. The homeostasis model assessment of insulin resistance (HOMA_IR_) was calculated as (fasting glucose (mM) × fasting insulin (mU/L))/22.5 [32].

### Statistical analysis

Sample size was determined *a priori *using nQuery Advisor (version 6.01, Statistical Solutions, Saugus, MA) with 85% power to detect a minimum difference in total weight loss of 3.3 kg between groups at study completion. Assuming a common SD of 3 kg and a 15-20% drop out rate, 23 subjects per group needed to complete the study. A sequence of random numbers without replacement was generated by computer algorithm to assign subjects to preload group [33].

For dietary data, RDs entered food and beverage items from the 24-hour recalls by unit weight into Nutrition Data System for Research software (NDS-R, version 2007, Nutrition Coordinating Center, University of Minnesota, MN). After entering recall data, RDs compared subjects' food logs to recall data to identify omissions in recalled intakes. Recipes were created for items not present in NDS-R using the gram weight of food ingredients consumed. Nutrient composition of created recipes was verified with food labels or *Bowes & Church's Food Values of Portions Commonly Used *[34]. Energy and nutrient intakes from all preloads, meals and snacks in each 24-hour period were combined to calculate total daily intakes.

Baseline descriptive characteristics for the sample were tested using Chi-square test of independence for categorical variables and one-way ANOVA for continuous variables. Chi-square tests of independence and Student's *t-*tests were used to compare dropouts to completers. Data were analyzed according to the intention-to-treat principle with last observation carried forward. Differential changes in outcome variables among the preload groups were tested using analysis of covariance (ANCOVA) with baseline values included as the covariate to control for possible baseline differences in outcome variables. Contrast analysis within ANCOVA was used to compare GF and GFJ groups to the water group. Relationships between changes (post-intervention minus baseline) in any two outcome variables were assessed using Spearman's correlation coefficients. Data were analyzed using SPSS software (version 15.0; SPSS Inc., Chicago, IL). Statistical significance was set at *p *< 0.05. Values are expressed as means ± standard deviation (SD).

## Results

### Subjects

Sixty-four women and 21 men completed the caloric restriction phase and were randomly assigned to GF, GFJ or water preloads. At baseline, there were no statistically significant differences according to preload assignment for age, gender, BMI, race, education or disordered eating scores (Table 2); subjects' average age was 38.7 ± 8.2 years and mean BMI was 35.6 ± 3.3 kg/m^2^. Seventeen subjects (20%) dropped out during study weeks 6-9. No significant differences in baseline characteristics were detected between dropouts and completers and no difference in attrition rates were observed by preload group (*p *= 0.94).

**Table 2:** Baseline Descriptive Characteristics of Subjects Randomized to Preload Group (n = 85)*

Characteristic	Grapefruit Group	Grapefruit Juice Group	Water Group
	(n = 29)	(n = 28)	(n = 28)
Completed Study (#, %)	23 (79.3%)	22 (78.6%)	23 (82.1%)
Gender			
Male	11 (37.9%)	3 (10.7%)	7 (25%)
Female	18 (62.1%)	25 (89.3%)	21 (75%)
Race			
Caucasian	13 (44.8%)	19 (67.9%)	19 (67.9%)
African American	16 (55.2%)	9 (32.1%)	9 (32.1%)
Education			
High School Degree	3 (10.3%)	3 (10.7%)	3 (10.7%)
Undergraduate Degree	15 (51.7%)	17 (60.7%)	21 (75.0%)
Graduate Degree	11 (37.9%)	8 (28.6%)	4 (14.3%)
Past Smoker^c^	2 (6.9%)	5 (17.9%)	3 (10.7%)
Age (years ± SD)^a^	37.6 ± 7.4	39.8 ± 8.4	38.7 ± 8.8
Height (cm ± SD)	165.9 ± 8.4	165.1 ± 6.4	166.9 ± 8.9
Weight (kg ± SD)	99.8 ± 13.8	95.9 ± 11.5	99.5 ± 13.5
Body Mass Index (mean ± SD)^b^	36.3 ± 3.1	35.2 ± 3.1	35.7 ± 3.5
Assessment of Eating Disorder			
EAT-26^d^	10.6 ± 6.4	10.4 ± 5.9	9.1 ± 5.9
Dietary Restraint Score^e^	10.5 ± 4.4	10.9 ± 3.5	10.7 ± 4.0
Disinhibition Score^e^	7.5 ± 2.7	8.2 ± 3.4	8.6 ± 3.0
Hunger Tendency Score^e^	5.3 ± 2.6	6.3 ± 3.2	6.1 ± 3.3
Depression History	1 (3.4%)	3 (10.7%)	2 (7.1%)
Metabolic Syndrome^f^	11 (37.9%)	6 (21.4%)	6 (21.4%)

a. Age 21-50 years required at study entry.

b. BMI 30-39.9 required to meet study eligibility.

c. Current (within past year) non-smoker required to meet study eligibility.

d. EAT-26 measures general eating disorder pathology; score ≥ 20 was criteria for study exclusion.

e. Three Factor Eating Questionnaire, dietary restraint score ≥ 14 was criteria for study exclusion.

f. Meeting ≥ 3 of 5 NCEP-ATPIII criteria.

* Demographic characteristics were not significantly different at baseline among randomly assigned preload groups based on Chi-square test of independence for categorical variables and one-way ANOVA for continuous variables.

### Weight loss and body composition

Subjects had an average weight loss of 0.99 ± 0.50 kg during the caloric restriction phase. The rate of weight loss increased significantly by 13.3% (*p *< .0001) during the caloric restriction + preload phase for an additional loss of 5.8 ± 3.9, 5.9 ± 3.6 and 6.7 ± 3.1 kg (GF, GFJ and water, respectively). Adjusted for baseline weight, total weight loss was not statistically different by group. As average weight loss across groups was 7.1% of initial body weight, BMI decreased significantly for all subjects (Table 3).

**Table 3:** Change in Outcome Variables from Baseline to Study Completion by Preload Group

	GF Preload (n = 29)	GFJ Preload (n = 28)	Water Preload (n = 28)	
	(mean ± SD)	(mean ± SD)	(mean ± SD)	P*
**Energy Expenditure**				
RQ (VCO_2_/VO_2_)	0.0 ± 0.1	0.0 ± 0.1	0.0 ± 0.1	0.618
REE (kcal)	4.5 ± 27.9	42.1 ± 18.4	- 37.1 ± 22.6	0.151
REE Adjusted (kcal/kg/lbm)	1.6 ± 4.6	1.7 ± 5.1	0.9 ± 5.2	0.078
**Body Composition**				
Body Mass Index (kg/m^2^)	- 1.6 ± 1.6	- 1.9 ± 1.4	- 2.1 ± 1.1	0.523
Waist Circumference (cm)	- 4.0 ± 4.1	- 5.5 ± 5.7	- 5.4 ± 4.8	0.189
Fat Tissue Mass (kg)	- 2.6 ± 2.1	- 2.9 ± 2.9	- 2.5 ± 2.1	0.499
Total Body Fat (%)	- 1.1 ± 1.8	- 1.1 ± 1.9	- 1.2 ± 2.6	0.489
Trunk Fat (%)	- 1.4 ± 2.9	- 1.7 ± 2.6	- 1.2 ± 2.6	0.154
Android Fat (%)	- 1.9 ± 2.4	- 1.2 ± 2.7	- 1.5 ± 3.3	0.239
Gynoid Fat (%)	- 1.5 ± 2.4	- 0.5 ± 2.9	- 0.7 ± 4.5	0.114
Lean Tissue Mass (kg)	- 0.9 ± 2.1	- 1.9 ± 1.9	0.3 ± 2.4	0.127
Lean Tissue Mass (%)	1.1 ± 2.4	0.8 ± 2.0.	1.8 ± 2.6	0.230
BMC (kg)	0.2 ± 0.2	0.0 ± 0.1	0.1 ± 0.2	0.587
**Glycemia and Blood Pressure**				
Fasting Glucose (mmol/L)	0.1 ± 0.3	0.1 ± .0.4	0.0 ± 0.4	0.969
Fasting Insulin (uU/mL)	- 0.5 ± 4.7	- 0.3 ± 3.7	- 0.8 ± 7.4	0.691
HOMA_IR _Score	- 0.2 ± 1.	- 0.1 ± 0.8	- 0.6 ± 1.6	0.095
Systolic Blood Pressure (mmHg)	- 3.1 ± 7.8	-3.1 ± 7.4	- 1.5 ± 6.3	0.922
Diastolic Blood Pressure (mmHg)	- 0.3 ± 8.1	- 3.8 ± 9.1	0.1 ± 8.1	0.565
**Lipids**				
Triglycerides (mg/dl)	- 6.7 ± 40.6	- 9.4 ± 31.9	- 4.3 ± 26.2	0.166
Total cholesterol (mg/dl)	3.0 ± 21.3	- 3.2 ± 14.9	2.5 ± 11.1	0.419
LDL cholesterol (mg/dl)	1.8 ± 3.2	5.3 ± 7.9	3.5 ± 7.7	0.498
HDL cholesterol (mg/dl)	3.0 ± 5.2	4.9 ± 7.5**	-2.0 ± 7.2	0.020
HDL to total cholesterol ratio	- 0.2 ± 0.4	- 0.4 ± 0.7**	0.2 ± 0.6	0.025

*P <. 0.05 indicating statistically significant difference by ANCOVA, with baseline value included as covariate. For outcomes showing significant differences by ANCOVA, simple contrasts were conducted within ANCOVA to determine which preloads were significantly different.

** Significantly different from water preload, *P*s = 0.017.

Weight loss significantly correlated with reduced waist circumferences (r = 0.37, *p *= 0.004) of 2.9 ± 4.1, 5.5 ± 5.7 and 5.4 ± 4.8 cm, respectively. Although there were statistically significant within-group decreases for waist circumference and percentage body, trunk, android and gynoid fat, there were no statistically significant differences among groups after adjusting for baseline values. Likewise, there were no statistically significant differences among groups in the change in the proportion of fat to lean mass.

### Energy balance and food intake

There were no statistically significant differences among groups at baseline or study completion for respiratory quotient, substrate oxidation rates, REE or REE adjusted for fat-free mass. Although pedometer counts indicated no difference among groups in steps walked daily, walking exercise capacity significantly improved for all groups with a mean change from 283 ± 3.5 to 269 ± 3.3 seconds (p < 0.001).

**Figure 2:**
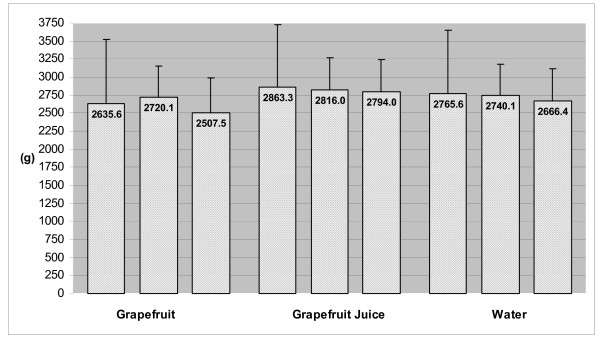
Total Amount of Food Consumed at Baseline (Week 0), End of Caloric Restriction Phase (Week 2) and End of Caloric Restriction + Preload Phase (Week 14)*. * Total Amount of Food = Average daily quantity of food consumed, includes dietary preloads (~127 g) during the caloric restriction + preload phase (week 14). Change in amount consumed not significantly different among preload groups based on ANCOVA.

**Figure 3:**
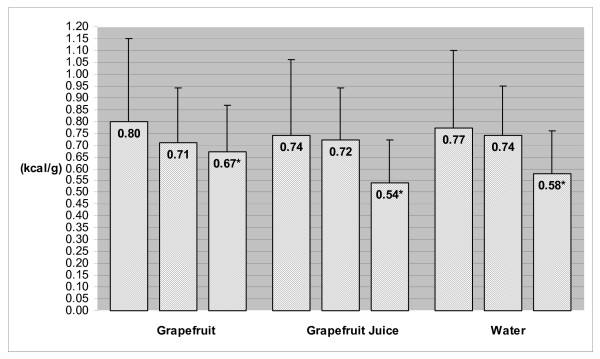
Dietary Energy Density at Baseline, End of Caloric Restriction Phase (Week 2) and End of Caloric Restriction + Preload Phase (Week 14)*. Dietary Energy Density = Average daily dietary energy density; includes GF, GFJ or water preload during the caloric restriction + preload phase (week 14). * Significantly different from baseline value based on ANCOVA with contrasts, *P *< 0.01.

**Figure 4:**
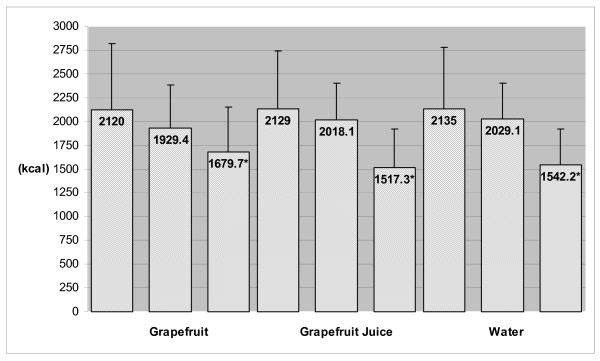
Total Energy Intakes at Baseline, End of Caloric Restriction Phase (Week 2) and End of Caloric Restriction + Preload Phase (Week 14)*. Total Energy Intakes = Average total daily energy intakes consumed; includes energy from dietary preloads (~46 kcal) during caloric restriction + preload phase (week 14). * Significantly different from baseline value based on ANCOVA with contrasts, *P *< 0.01.

Baseline (habitual) and prescribed (16.6 ± 0.3, 16.4 ± 0.2 and 16.5 ± 0.3 kcal/kg; GF, GFJ and water, respectively) energy intakes did not differ among groups. As displayed in Figure 2, there were no significant changes over time in the average amount (grams) of total food consumed daily. During caloric restriction phase, average reported total energy intakes decreased by 9% in GF group, 5% in GFJ group and 5% in water group. However, when preloads were combined with caloric restriction, average dietary energy density decreased by 27.9% in GF group, 21.6% in GFJ group and 20.3% in water group (Figure 3) and average total energy intakes decreased by 21% in GF group, 29% in GFJ group, and 28% in water group (Figure 4). After adjustment for baseline values, the differences among groups in dietary energy density and total energy intakes were not statistically significant.

Likewise, there were no significant differences among groups at baseline or over the course of the study for total fluid intakes or macronutrient intakes (as percentages of energy). Total dietary fiber intake was significantly increased in the GF group (by 3.4 ± 1.5 g/d) compared to water group (*p *= 0.030), which demonstrates compliance with consuming GF preloads. Similarly, average vitamin C intakes were significantly increased in GF and GFJ (by 130.5 ± 62.8 and 137.3 ± 46.7 mg/d, respectively) compared to water group (*p *< 0.001). There were no significant changes in mean VAS ratings for hunger, thirst, satiety or fullness, but VAS ratings for appetite significantly decreased in the GFJ group from 80 ± 4 to 58 ± 6 mm, *p *= 0.002.

### Dyslipidemia and cardiometabolic risk

The mean changes in total and LDL cholesterol did not differ significantly from baseline. In contrast, within-group triglyceride levels decreased significantly, but these changes did not differ by group when adjusted for baseline values. The primary difference among groups was the amount and direction of change in serum HDL-C concentration and total:HDL-C ratio. There was a mean increase in HDL-C from baseline by 6.2% in the GF group and 8.2% in the GFJ group - which differed significantly from the mean decrease of 3.7% in the water group (*p *= 0.003 and 0.009, respectively).

There were no significant changes from baseline detected in blood pressure, fasting glucose, insulin and HOMA scores, perhaps a reflection of baseline and study completion values that were within normal ranges. Overall, the proportion of subjects who met criteria for metabolic syndrome significantly decreased from 27% at baseline to 20% at study completion, *p *< 0.001.

## Discussion

This study is one of few randomized trials comparing the effects of consuming low energy dense preloads as part of a dietary weight loss intervention in free-living obese adults. The study is unique because: 1) we utilized solid and liquid forms of a fruit preload that were matched for weight, energy, water contents, and thus, energy density; 2) GF and GFJ preloads were compared to a water preload matched by weight (127 g) since the composition of GF and GFJ is ~91% water; and 3) preloads were ingested 20 minutes before meals to avoid potential confounding effects of orogastrointestinal satiety signaling [27]. Thus, any differential responses to the preload strategy would result from the higher fiber content of GF or higher bioflavonoid content of GFJ.

Under these conditions, dietary energy density reduced 20-28% and total energy intakes decreased 21-29% after preloads were incorporated into the meal plan. Interestingly, reduced energy intakes were not associated with higher VAS ratings of hunger, indicating that subjects remained satiated [35]. If the amount (grams) of food consumed is a determinant of hunger [36], the lack of perceived hunger may be explained by the consistent amount of food consumed throughout the study. It is intriguing that subjects not only adjusted the total amount of their food intakes to incorporate the amount of the preloads, but also compensated for the energy content of the preloads by decreasing energy intakes from meals and snacks to achieve an overall reduction in total energy intakes.

The reduction in dietary energy density and energy intakes achieved represents an 8.5-16.5% (~250-500 kcal/d) greater reduction in calories consumed during the caloric restriction + preload phase than the 12.5% reduction prescribed during the caloric restriction phase. This finding is consistent with other community-based interventions in which consumption of a low energy dense diet has led to substantial reductions in energy intakes and body weight [37-40]. In the present study, while the overall weight loss of 7.1% of initial body weight was not statistically different among groups, weight loss was clinically meaningful based on current consensus that 5-10% weight loss decreases cardiometabolic risk [41].

Notably, the additional 8.5-16.5% reduction in energy intakes during the caloric restriction + preload phase was physiologically consistent with the 13.3% increase in the rate of weight loss during that phase. The compensation observed contrasts with some basic science models of energy balance utilizing the concept of negative adiposity feedback signaling to the brain [42-44] and data suggesting that obese individuals would defend adiposity and compensate for weight loss by increasing intakes of energy dense foods or total calories [42,44,45]. Nevertheless, our findings are consistent with the ability of individuals at lower BMI to respond to the energy content of an ingested preload [11]. That our obese subjects exhibited such a response in the setting of negative energy balance and weight loss suggests that utilization of a low energy dense preload may fundamentally influence mechanisms involved in energy homeostasis [37,46].

The present data indicate that preload weight and low energy density, not form (solid *vs *liquid), fiber or bioflavonoid content promoted the greater reductions in dietary energy density, total energy intakes, and body weight. While this contrasts with laboratory-based experiments that show differential effects on energy intakes at a meal based on the physical form of food [7,8,12,13,47,48], it is consistent with data outside of the lab setting where subjects who logged 24-hour food diaries showing no differences in total energy intakes when consuming solid and liquid preloads of several different food items [49].

It is also intriguing that the water preload was equally efficacious for reducing energy intakes and body weight. This finding also suggests that it was preload consumption that affected dietary energy density and total energy intakes during the caloric restriction + preload phase. Since water adds weight (and volume) without energy, increasing the amount of water in a food or beverage item is a common method for manipulating energy density [36,49-51] and incorporating water into beverage, soup and casserole preloads has reduced subsequent lunch meal energy intakes by 7-20% [5,6,50]. In addition, subjects who drank water with breakfast reported less hunger and greater satiety [52], and when drinking water replaced caloric beverages energy intakes decreased and subjects lost weight [53].

Though improvements in insulin sensitivity and lipoprotein profile frequently occur during weight loss [54], we did not detect significant changes in glucose, insulin, or total and LDL-cholesterol. A plausible explanation is that the small changes observed reflect a low level of insulin resistance in these relatively healthy obese subjects. It is striking that HDL-C levels increased up to 8.2% from baseline in GF and GFJ groups, a significant change compared to decreased HDL-C in the water preload group. Since epidemiological evidence indicates that raising HDL-C by only 1 mg/dL reduces cardiovascular risk by 2-3%, this finding supports earlier evidence of potential anti-atherosclerotic effects of GF or GFJ consumption. A possible explanation for the rise in HDL-C is increased antioxidant activity from greater vitamin C and/or flavonoid (ie, naringin) intakes [55,56], although oxidative stress was not directly measured in the present study.

While the present study was carefully designed to compare the effects of GF, GFJ and water preloads, limitations are worth considering. In contrast to laboratory-based feeding, it was not possible to blind study RDs and subjects to preload assignment in this community-based dietary intervention. Second, there is no food or beverage that functions as a completely inactive comparator as even water may have metabolic effects under certain conditions [57,58]. Yet, the high (~91%) water content of GF and GFJ made the water preload an appropriate control for analytic comparisons. Moreover, including water allowed all groups to experience similar behaviors and orogastric sensations while preloading three times daily for 12 weeks. Third, while we acknowledge that obese adults usually underreport energy intakes [59], the potential for underreporting should be equivalent among subjects as there were no differences by group in baseline BMI [60]. Even so, to compensate for potential bias, RDs conducted unannounced randomly scheduled 24-hr recalls by telephone using validated methods and standardized scripts [61,62]. Further, subjects were trained to estimate portion sizes using visual aids designed to improve recall accuracy [63]. Additionally, our 24-hr recall and food log data agreed with the expected changes in dietary energy, fiber and vitamin C intakes. These improvements in nutrient intake profiles indicate reliable reporting as well as evidence of high compliance with the dietary protocol.

## Conclusions

Our findings complement the accumulating body of evidence demonstrating that clinically significant weight loss can be achieved when consuming a low energy dense preload before meals. Notably, we demonstrate that this type of dietary intervention can occur without decreasing the total amount of food consumed, and thus, without inducing the hunger and dissatisfaction often associated with restrictive diets. Compared to pharmacological trials in free-living obese adults where attrition rates range from 30-40% [64], we achieved a high completion rate (80%), further indication that subjects found the preload strategy satisfying, and they may have gained intrinsic value from interactions with study RDs.

Further, we extend the evidence by showing that the preload strategy can reduce dietary energy density and total energy intakes in obese adults in free-living conditions regardless of the form of the preload. Obese individuals such as these might be more inclined to utilize the concept of "volumetrics" [65] if encouraged to choose the form of their low energy dense preload based on individual preference. Our data supports such choice in the context of a dietary weight loss intervention and suggests that such interventions have a fundamentally physiological basis.

Importantly, the bioactive components of some preloads, like GF and GFJ, may confer additional cardiometabolic benefits as evidenced by the very significant increases in serum HDL-cholesterol concentrations in the present trial. Clearly, additional clinical research is needed to investigate the mechanisms by which fruit, juice and water influence energy intake regulation and lipid metabolism.

## Competing interests

The authors declare that they have no competing interests.

## Authors' contributions

HJS obtained primary funding for the study, designed and conducted the study, participated in statistical analysis, and conceived of and wrote the article. MSD performed power and sample size calculations, designed the randomization and statistical analysis plan, and performed statistical analyses. KDN participated in evaluation of study findings, development of the article, and revised the article for important intellectual content. All authors read and approved the final manuscript.

## Authors' information

Heidi J. Silver, Ph.D., R.D is the Research Assistant Professor of Medicine at the Vanderbilt University School of Medicine, Nashville, TN. Mary S. Dietrich, Ph.D. is the Research Associate Professor of Nursing and Medicine at the Vanderbilt University Schools of Nursing and Medicine, Nashville, TN. Kevin D. Niswender, M.D., Ph.D is Assistant Professor of Medicine at the Vanderbilt University School of Medicine, Nashville, TN.
